# SMP30-mediated synthesis of vitamin C activates the liver PPARα/FGF21 axis to regulate thermogenesis in mice

**DOI:** 10.1038/s12276-022-00888-9

**Published:** 2022-11-25

**Authors:** Bonggi Lee, Hye Jin An, Dae Hyun Kim, Min-Kyeong Lee, Hyeon Hak Jeong, Ki Wung Chung, Younghoon Go, Arnold Y. Seo, Il Yong Kim, Je Kyung Seong, Byung Pal Yu, Jaewon Lee, Eunok Im, In-Kyu Lee, Myung-Shik Lee, Ken-ichi Yamada, Hae Young Chung

**Affiliations:** 1 Department of Food Science and Nutrition, Pukyong National University, Daeyeon-dong, Nam-gu, Busan, South Korea; 2 Department of Pharmacy, College of Pharmacy, Pusan National University, Busan, 46241 South Korea; 3 Molecular Inflammation Research Center for Ageing Intervention (MRCA), Pusan National University, Busan, 46241 South Korea; 4 Department of Smart Green Technology Engineering, Pukyong National University, Daeyeon-dong, Nam-gu, Busan, 48513 South Korea; 5 Korean Medicine Application Center, Korea Institute of Oriental Medicine, Daegu, South Korea; 6 Janelia Research Campus, Howard Hughes Medical Institute, Ashburn, VA USA; 7 Laboratory of Developmental Biology and Genomics, Research Institute for Veterinary Science, and BK21 Plus Program for Creative Veterinary Science, College of Veterinary Medicine, Seoul National University, Seoul, South Korea; 8 Korea Mouse Phenotyping Center (KMPC), Seoul National University, Seoul, South Korea; 9 Interdisciplinary Program for Bioinformatics, Program for Cancer Biology and BIO-MAX Institute, Seoul National University, Seoul, South Korea; 10 Department of Physiology, The University of Texas Health Science Center at San Antonio, San Antonio, TX USA; 11 Department of Internal Medicine, Kyungpook National University School of Medicine, Daegu, South Korea; 12 Severance Biomedical Science Institute and Department of Internal Medicine Yonsei University College of Medicine, Seoul, South Korea; 13 Department of Bio-functional Science, Kyushu University, Fukuoka, Japan

**Keywords:** Transgenic organisms, Fat metabolism

## Abstract

The vitamin-C-synthesizing enzyme *senescent marker protein 30* (SMP30) is a cold resistance gene in Drosophila, and vitamin C concentration increases in brown adipose tissue post-cold exposure. However, the roles of SMP30 in thermogenesis are unknown. Here, we tested the molecular mechanism of thermogenesis using wild-type (WT) and vitamin C-deficient SMP30-knockout (KO) mice. SMP30-KO mice gained more weight than WT mice without a change in food intake in response to short-term high-fat diet feeding. Indirect calorimetry and cold-challenge experiments indicated that energy expenditure is lower in SMP30-KO mice, which is associated with decreased thermogenesis in adipose tissues. Therefore, SMP30-KO mice do not lose weight during cold exposure, whereas WT mice lose weight markedly. Mechanistically, the levels of serum FGF21 were notably lower in SMP30-KO mice, and vitamin C supplementation in SMP30-KO mice recovered FGF21 expression and thermogenesis, with a marked reduction in body weight during cold exposure. Further experiments revealed that vitamin C activates PPARα to upregulate FGF21. Our findings demonstrate that SMP30-mediated synthesis of vitamin C activates the PPARα/FGF21 axis, contributing to the maintenance of thermogenesis in mice.

## Metabolism: Vitamin C synthesis enzyme linked to heat production

An enzyme involved in vitamin C synthesis also mediates the ability of mice to generate heat in response to cold exposures. Hae Young Chung from Pusan National University in Busan, South Korea, and colleagues showed that mice without a working version of this enzyme, known as SMP30, do not lose weight under cold conditions. That’s because they cannot burn brown fat to create heat through a process called thermogenesis. The researchers detailed how vitamin C, whether produced naturally by SMP30-related signaling in mouse tissues or given via dietary supplement, triggers the activation of signaling molecules and growth factors necessary for thermogenesis. Since humans cannot synthesize their own vitamin C, and thus cannot take advantage of this heat-generating and fat-burning pathway, these findings may help explain a common cause of weight gain and obesity.

## Introduction

Senescent marker protein 30 (SMP30) or regucalcin was characterized by two different groups as a gluconolactonase for vitamin C synthesis^1^ and a calcium-binding protein^2^, respectively. SMP30 is evolutionally conserved in different species, including humans, rats, mice, and Drosophila^3–5^. The gluconolactonase function of SMP30 is found in various animal species and is essential for producing L-gulono-γ-lactone, an important precursor for vitamin C (L-ascorbic acid) synthesis^6^. Therefore, SMP30-knockout (KO) mice are deficient in vitamin C, and they start to lose weight from approximately 11 weeks of age^7^ and die within approximately 20 weeks after starting a vitamin C-deficient diet^1^. Histopathological analysis showed no apparent reason for death except emaciation. Thus, SMP30-KO mice are considered an aging mouse model that exhibits wasting syndrome related to aging^1,6^.

Although having SMP30, humans cannot synthesize vitamin C due to genetic mutations in L-gulono-γ-lactone oxidase, which is the SMP30 subsequent enzyme for vitamin C synthesis from L-gulono-γ-lactone. Some human studies have shown that vitamin C status is negatively associated with the onset of obesity^8,9^. Therefore, it is suggested that the inability to synthesize vitamin C in humans may be closely associated with the current obesity epidemic^10^.

Vitamin C is a well-known reducing agent and antioxidant that is not present in significant amounts in body stores. A few species, including humans, cannot synthesize vitamin C endogenously and therefore require supplementation in the diet to prevent scurvy^11^. Despite multiple studies, there is no consensus regarding its usefulness in energy metabolism^12,13^. Nevertheless, several studies have suggested that vitamin C levels are negatively associated with body weight. Vitamin C levels are markedly lower in obese individuals than in lean subjects and, thus, are negatively correlated with body mass index^8,9,14–16^. A high dose of vitamin C (3 g/day) expedites weight loss in severely obese individuals^17^. Furthermore, vitamin C deficiency was reported in 35–45% of severely obese individuals planning to undergo bariatric surgery^18^.

Although the mechanism underlying vitamin C-mediated regulation of energy balance is still ill-defined, some studies have suggested a potential role of vitamin C in controlling body temperature. For example, a clinical trial reported that vitamin C supplementation for 17 days helps healthy subjects maintain a warmer body temperature in the winter^19^. In addition, the oral administration of vitamin C significantly increases body temperature in humans and guinea pigs, neither of which endogenously produce vitamin C^20,21^. However, no studies have investigated the roles of the vitamin C-synthesizing enzyme SMP30 and the endogenous synthesis of vitamin C in thermogenic energy expenditure. Based on these observations, we hypothesized that SMP30-mediated endogenous synthesis of vitamin C is necessary for the thermogenic activation of adipose tissues. We investigated the physiological and molecular mechanisms by which SMP30 deficiency affects energy balance, especially via the regulation of thermogenesis, using multiple mouse and cell models.

## Materials and methods

### Mouse models

For the SMP30-KO mouse studies, C57BL/6 background SMP30-KO mice were kindly provided by Dr. Akihito Ishigami (Tokyo Metropolitan Institute of Gerontology, Tokyo 173-0015, Japan). Their genotypes were confirmed in our laboratory using a previously described method^22^. Because SMP30-KO mice are vitamin C-deficient, they were provided drinking water that included 1.5 g/L vitamin C before starting the metabolic experiments to prevent vitamin C deficiency-mediated early death^7^. To investigate the changes in energy balance, SMP30-KO mice were given water without vitamin C 2 weeks before starting experiments to deplete vitamin C. The 10–11-week-old male WT and SMP30-KO mice were fed a chow diet or a high-fat (HF) diet for 3 weeks. All mice were maintained at 23 ± 2 °C with a relative humidity of 60 ± 5% and a 12-h light-dark cycle. They had free access to water and the HF diet. All animal studies were approved by the Institutional Animal Care Committee of Pusan National University. The study adhered to all guidelines for animal experiments issued by Pusan National University (Approval Number PNU-2016-1084).

### Cold challenge tests

For the acute cold challenge test, overnight-fasted WT, SMP30-KO, and SMP30-KO mice given vitamin C water (2 g/L) were maintained in a cold room (6 °C) for 5 h. During cold exposure, to minimize stress-induced increases in temperature, the cutaneous temperature was measured using an infrared thermometer for small rodents (BIO-IRB153; Bioseb, Vitrolles Cedex, France) because there is a strong correlation between cutaneous and core body temperatures, as measured by telemetry and rectal probes^23^. To ensure accurate estimates, the temperature was measured at multiple sites in the abdominal region of mice, and average values were obtained. For the prolonged cold challenge test, nonfasted WT, SMP30-KO, and SMP30-KO mice given vitamin C water were maintained in a cold chamber (6 °C) for 48 h, which induced adipose tissue browning based on our preliminary experiments. Vitamin C water (6 g/L; 400 µL) was administered to SMP30-KO mice by oral gavage three times a day (~7 mg vitamin C/mouse/day) to ensure a sufficient vitamin C supply during prolonged cold exposure.

### Indirect calorimetry

On Days 1–4, mice were acclimated in a metabolic chamber at room temperature (22 °C). On Day 5, oxygen consumption (VO_2_), carbon dioxide production (VCO_2_), and the respiratory exchange ratio (RER) were measured at 22 °C for 3 days using an indirect calorimetry system (PHENOMASTER, TSE Systems, Bad Homburg, Germany). Locomotor activity was determined at the same time that energy expenditure was measured using infrared beam interruption. The values of VO_2_ and VCO_2_ were normalized by the lean body mass. Lean body mass was measured by ^1^H magnetic resonance spectroscopy (Bruker BioSpin).

### Western blotting

Mechanically homogenized tissue samples were boiled for 10 min in gel-loading buffer (125 mM Tris-HCl, 4% sodium dodecyl sulfate [SDS], 10% 2-mercaptoethanol, pH 6.8, 0.2% bromophenol blue). Total protein equivalents for each sample were separated by SDS–polyacrylamide gel electrophoresis and transferred to PVDF membranes. Immunoblotting was performed with antibodies against SMP30 (a kind gift from Tokyo Metropolitan Geriatric Hospital and Institute of Gerontology), UCP1 (SC-6529; Santa Cruz Biotechnology, Dallas, TX, USA), β-actin (SC-47778; Santa Cruz), GAPDH (SC-32233; Santa Cruz), TFIIB (SC-271736; Santa Cruz), PPAR-α (ab24509; Abcam), and FGF21 (AF3057; R&D Systems, Minneapolis, MN, USA or SC 292879). Western blots were visualized by enhanced chemiluminescence (Davinch-Chemi System, CAS400) according to the manufacturer’s instructions. The SMP30 antibody was validated using liver samples from WT and SMP30-KO mice.

### Quantitative real-time PCR

RNA was isolated from inguinal adipose tissue, interscapular brown adipose tissue, Ac2F rat hepatocytes (Japanese Collection of Research Bioresources Cell Bank, JCRB0408), and primary adipocytes by using TRIzol (Invitrogen, Carlsbad, CA, USA). cDNA was synthesized using the SensiFAST cDNA Synthesis Kit (BIO-650531; Bioline, London, UK). Quantitative real-time PCR was performed using the SYBR SensiFAST qPCR Kit (BIO-98005; Bioline) and the CFX Connect Real-time PCR System (Bio-Rad, Hercules, CA, USA). All primer sequences were designed based on previous publications^24,25^.

### Vitamin C measurement

The vitamin C-specific fluorophore-nitroxide probe Naph-DiPy nitroxide (a kind gift from Dr. Ken-ichi Yamada, Department of Bio-functional Science at Kyushu University) was used to measure serum vitamin C concentrations^26^. Briefly, 5 μL of a serum sample was added to an assay buffer including 85 μL of distilled water and 10 μL of probe stock (500 μM in DMSO). The fluorescence intensity was determined at excitation and emission wavelengths of 310 and 430 nm, respectively.

### Analysis of circulating hormones

Blood was collected from mouse hearts under fed or overnight-fasted (from 7:00 pm to 12:00 pm) conditions, and serum samples were prepared by centrifugation (4 °C) at 2000 × *g* for 15 min. Blood FGF21 was measured using a Mouse/Rat FGF-21 Quantikine ELISA Kit (MF-2100; R&D Systems). Serum glucose, cholesterol, triglycerides, and nonesterified fatty acids were analyzed using kits from Bioassay Systems (Hayward, CA, USA).

### Luciferase analysis

For the PPARα or FGF21 luciferase assay, 1.5 × 10^4^ Ac2F cells/well were grown in a 96-well plate in DMEM supplemented with 10% FBS. The peroxisome proliferator response element (PPRE)-X3-TK-LUC plasmid (0.1 μg) (a kind gift from Dr. Christoper K. Glass, University of California, San Diego, CA, USA) and 0.01 μg of PPARα expression vector or pGL3B-Fgf21-LUC were transfected with Lipofectamine 3000 (0.1 μL) and P3000 (0.2 μL, Invitrogen) complexes in Opti-MEM (Invitrogen) according to the manufacturer’s instructions. The pcDNA empty vector was added to obtain an equal amount of plasmid DNA per transfection. After 24 h of transfection, cells were treated with vitamin C. Luciferase activity was tested using the ONE-Glo Luciferase Assay System (Promega, Madison, WI, USA) and a luminescence plate reader (Berthold Technologies GmbH & Co., Bad Wildbad, Germany).

### Statistical analysis

The sample size was determined with adequate power based on the literature and our previous studies. We performed pilot animal studies to obtain sample means and standard deviations (SDs)^19^. Using the sample means and SDs from the pilot studies, we predicted the necessary sample size based on the method provided in “http://www.lasec.cuhk.edu.hk/sample-size-calculation.html”. Data are expressed as the mean ± SEM unless otherwise indicated. GraphPad Prism 5.0 (GraphPad Software, San Diego, CA, USA) was used for Student’s *t-*tests (two-tailed) and ANOVA followed by Bonferroni post hoc tests (see figure legends for the statistical information for each figure). Differences were considered significant at *P* < 0.05.

## Results

### Vitamin C-deficient SMP30-KO mice exhibit lowered energy expenditure and body temperatures

**Fig. 1:**
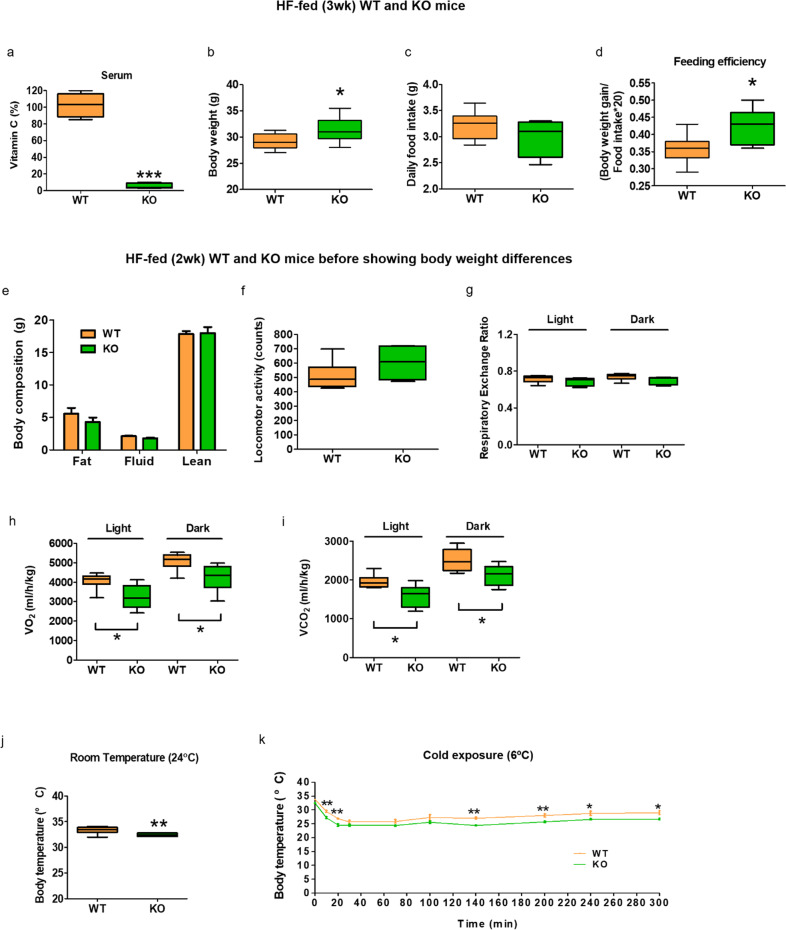
Increased body weight in SMP30-KO mice is correlated with reduced energy expenditure. Male SMP30-KO mice (10–11 weeks old) were fed an HF diet for 3 weeks (*n* = 7–8/group). **a** Serum vitamin C was measured using the fluorophore-nitroxide probe Naph-DiPy nitroxide. **b** Body weight was measured at 3 weeks of HF feeding. **c** Food intake was determined every other day for 2 weeks. **d** Feeding efficiency (the ratio of body weight to energy intake × 20) was measured at 2 weeks of feeding with an HF diet. For indirect calorimetry, 2-week HF-fed male mice (*n* = 6/group), before significant differences in body weight were observed, were used to measure **e** body composition, **f** locomotor activity, **g** respiratory exchange ratio, **h** O_2_ consumption, and **i** CO_2_ production. **j** Mouse body temperature at room temperature (24 °C) and **k** the changes in body temperature during acute cold exposure (6 °C for 5 h) were determined using an infrared thermometer for small rodents (*n* = 7–8/group). Data are presented as the mean ± SEM. *P < 0.05, **P < 0.01, and ***P < 0.001 for WT vs. SMP30-KO mice. Two-tailed Student’s t-test (**a**–**j**) and two-way ANOVA followed by Bonferroni post hoc tests (**k**). WT, wild-type mice; KO, SMP30-KO mice.

To examine the roles of SMP30 in energy metabolism, we used SMP30-KO mice, which cannot produce vitamin C owing to a lack of its precursor l-gulono-1,4-lactone. Because prolonged vitamin C deficiency causes severe weight loss^7^ and death^1^, we performed a short-term HF feeding study to observe changes in energy balance. We fed 10–11-week-old wild-type and SMP30-KO mice a vitamin C-deficient HF diet (60% of calories as fat) for 3 weeks. In this experimental condition, the symptoms of global vitamin C deficiency were not observed, which is consistent with other studies^1,27^. Compared to WT mice, blood vitamin C was largely depleted in the HF diet-fed SMP30-KO mice when measured at the end of the study (Fig. 1a), suggesting that the WT, but not SMP30-KO mice, endogenously synthesize vitamin C. The body weight of HF-fed SMP30-KO mice was higher than that of WT mice (Fig. 1b). To investigate the mechanisms underlying the altered body weight in SMP30-KO mice, we examined feeding behavior. There was no significant difference in daily food intake assessed over 2 weeks (Fig. 1c), but the feeding efficiency was notably higher in SMP30-KO mice than in WT mice (Fig. 1d). Increased feeding efficiency was also found in SMP30-KO mice after chronic HF feeding (3 months), although there were no significant differences in body weight (Supplementary Fig. 1a, b), possibly reflecting the effects of prolonged vitamin C deficiency. The consistently increased feeding efficiency during both acute and chronic HF feeding impelled us to measure energy expenditure by indirect calorimetry. To test whether altered energy expenditure is the main mechanism underlying the increase in body weight of the mice deficient in vitamin C, 2-week HF-fed male mice, a timepoint before significant differences in body weight are observed, were used to measure parameters related to energy expenditure (Fig. 1e). O_2_ consumption and CO_2_ production were lower in SMP30-KO mice than in WT mice during both the light and dark cycles, without differences in locomotor activity and the respiratory exchange ratio (Fig. 1f–i), indicating that the lower energy expenditure contributes to the higher body weight in SMP30-KO mice.

We further determined whether the altered energy expenditure is related to changes in body temperature. We measured body temperature under basal and cold conditions using an infrared thermometer for small rodents. SMP30-KO mice had slightly but significantly reduced temperatures under basal conditions (24 °C) (Fig. 1j). To investigate the effect of SMP30 deficiency on adaptive thermogenesis, mice were exposed to cold (6 °C) for 5 h, and their temperature was monitored. Compared to that of WT mice, the temperature of SMP30-KO mice was maintained at lower levels over the entire experimental period (Fig. 1k). These results suggest that SMP30 plays an important role in thermogenesis.

### SMP30-KO adipose tissues exhibit impaired thermogenic programming

**Fig. 2:**
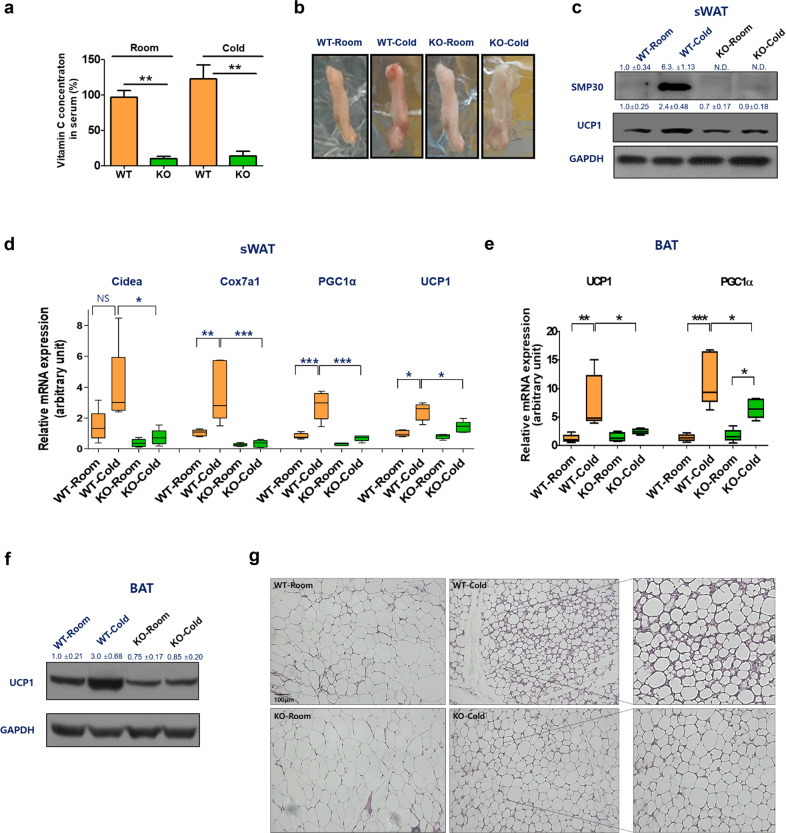
Impaired thermogenic activation of sWAT and BAT in SMP30-KO mice. To observe the changes in the protein levels of SMP30 and UCP1 before and after cold stimulation, acute cold exposure (6 °C for 5 h) was applied to chow diet-fed WT and SMP30-KO mice after overnight fasting. **a** Serum vitamin C levels were measured using the fluorophore-nitroxide probe Naph-DiPy nitroxide. **b** sWAT (inguinal fat) images were obtained immediately after dissection. **c** Representative images of western blots showing the protein levels of SMP30 and UCP1 in the sWAT of WT and SMP30-KO mice (*n* = 3–4/group). qPCR was performed to determine the mRNA expression levels of **d** WAT browning marker genes or **e** BAT activation-related genes (*n* = 5–6/group). **f** Representative images of western blots showing the protein levels of UCP1 in the BAT of WT and SMP30-KO mice (*n* = 3–4/group). To further examine the changes in adipose tissue morphology, prolonged cold exposure (6 °C for 48 h) was applied to chow diet-fed WT and SMP30-KO mice (*n* = 6–8/group) without fasting. **g** Representative H&E-stained images (four total images per group) showed prolonged cold exposure-induced alterations in sWAT morphology. Data are expressed as the mean ± SEM. *P < 0.05, **P < 0.01, and ***P < 0.001. One-way ANOVA followed by Bonferroni post hoc tests (**c**, **d**).

Recent studies have established that WAT browning and BAT activation are responsible for adaptive thermogenesis^24^. To investigate whether WAT browning is impaired in SMP30-KO mice deficient in vitamin C, the mice were maintained in a cold chamber at 6 °C for 5 h, which induces WAT browning based on our preliminary experiments. Serum vitamin C levels were measured before and after cold exposure. Although it was not significant, the serum vitamin C level trended to be higher after cold exposure in WT mice, but no changes were observed in SMP30-KO mice before and after cold exposure (Fig. 2a). A cold-induced change in sWAT color was not observed in SMP30-KO-sWAT (Fig. 2b). Western blotting further revealed that WT-sWAT exhibited increased protein levels of SMP30 and UCP1 after cold exposure, whereas these responses were not observed in SMP30-KO-sWAT (Fig. 2c). Consistent with these results, cold exposure upregulated the expression of browning markers, including *Ucp1*, *Cidea*, *Cox7a1*, and *Pgc1α*, in WT-sWAT, but these characteristics were impaired in SMP30-KO-sWAT (Fig. 2d). Similar to sWAT, cold exposure notably increased both the protein and mRNA levels of *Ucp1* and *Pgc1α* in WT-BAT, and the expression levels of these genes were reduced in SMP30-KO-BAT (Fig. 2e and f). To further examine the changes in adipose tissue morphology, prolonged cold exposure (6 °C for 48 h) was applied to chow diet-fed WT and SMP30-KO mice. H&E staining revealed that in response to cold conditions, WT-sWAT displayed greatly increased multilocular lipid droplets, a characteristic of BAT, and this response was disrupted in SMP30-KO-sWAT (Fig. 2g). These data demonstrate that cold-mediated WAT browning and BAT activation were impaired in vitamin C-deficient SMP30-KO mice.

### Thermogenic hormone FGF21 production is disrupted in SMP30-KO mice

**Fig. 3:**
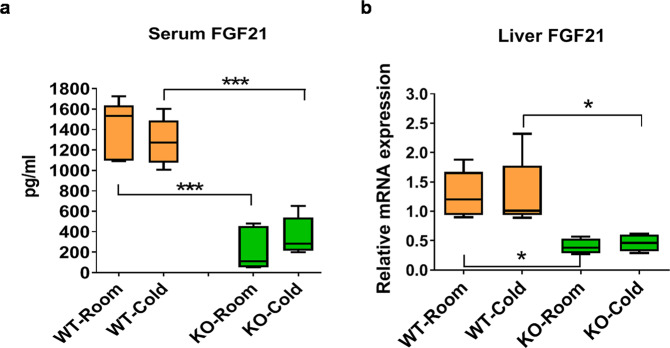
Impaired FGF21 production in SMP30-KO mice deficient in vitamin C. To investigate whether the SMP30/vitamin C axis regulates the thermogenic hormone FGF21 before and after cold exposure (cold exposure after overnight fasting), **a** serum FGF21 levels (*n* = 5/group) and **b** *Fgf21* mRNA expression levels in the liver (n = 5/group) were measured using an ELISA kit and qPCR, respectively. Serum FGF21 data were repeated in two cohorts of mice (total *n* = 15–17/group). Data are expressed as the mean ± SEM. *P < 0.05 and ***P < 0.001. One-way ANOVA followed by Bonferroni post hoc tests.

It has been reported that vitamin C ameliorates perfluorooctane sulfonate-induced hepatic steatosis and elevates FGF21 levels in circulation^28^. Because FGF21 is a thermogenic hormone that is mainly secreted from the liver, we studied whether vitamin C regulates FGF21 in normal and cold conditions without other treatments. Compared to WT mice, blood FGF21 levels were much lower in SMP30-KO mice housed at both room (24 °C) and cold temperatures (6 °C) (86 and 72% reductions, respectively), but no cold-induced changes were detected in blood FGF21 (Fig. 3a). In the liver, *Fgf21* mRNA levels significantly decreased in SMP30-KO mice regardless of cold exposure (Fig. 3b). These data demonstrate that FGF21 production is impaired under vitamin C-deficient conditions regardless of cold exposure. In addition, the marked decrease in FGF21 in the blood and liver is associated with the reduced thermogenesis and energy expenditure observed in SMP30-KO mice.

### Vitamin C supplementation recovers the impaired FGF21 production and adipose tissue browning in SMP30-KO mice

**Fig. 4:**
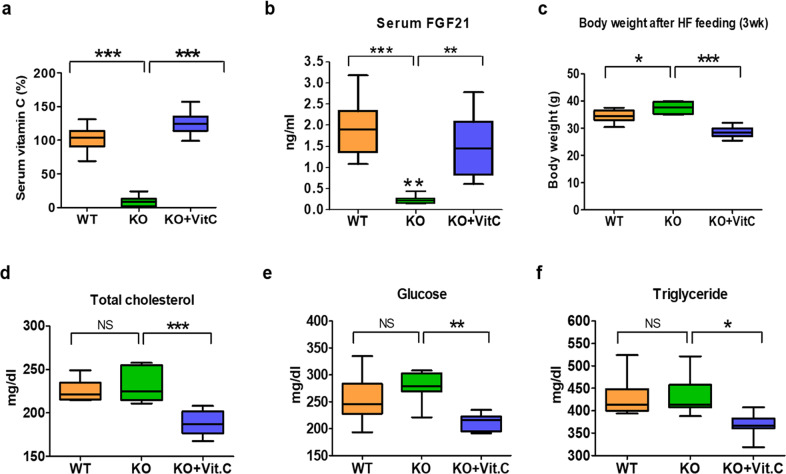
Vitamin C supplementation reverses the increased body weight in SMP30-KO mice. Male mice were fed an HF diet for 3 weeks (*n* = 7–8/group). **a** Serum vitamin C and **b** FGF21 levels were measured after overnight fasting using the fluorophore-nitroxide probe Naph-DiPy nitroxide and an ELISA kit, respectively. **c** Final body weight of WT, SMP30-KO, and SMP30-KO mice supplemented with vitamin C (2 g/L). **d** Total cholesterol, **e** glucose, and **f** triglycerides in serum were measured after overnight fasting by enzymatic colorimetric assay kits. Data are presented as the mean ± SEM. *P < 0.05, **P < 0.01 and ***P < 0.001. One-way ANOVA followed by Bonferroni post hoc tests (**a**–**f**).

**Fig. 5:**
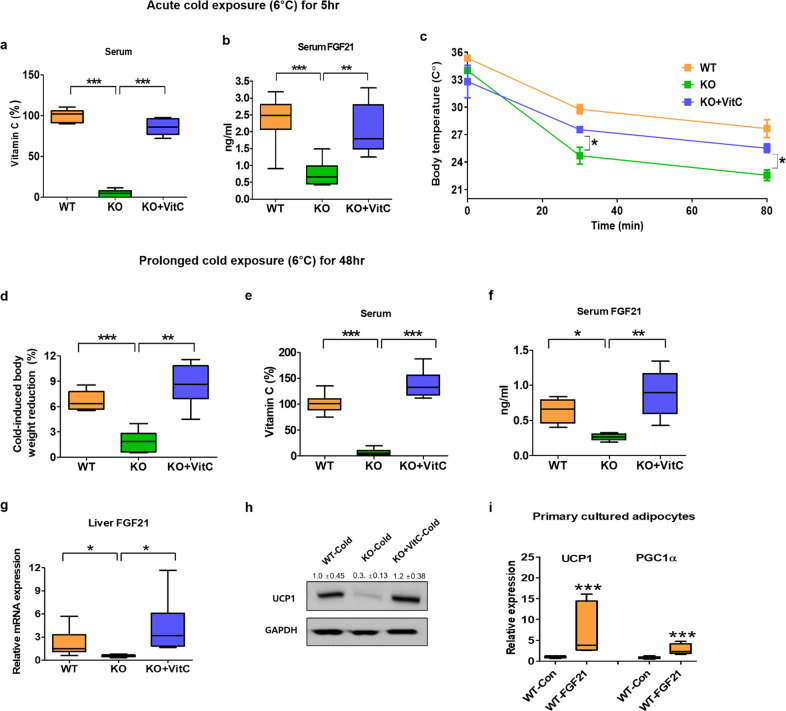
Vitamin C treatment recovers the impaired FGF21 production and body temperature in SMP30-KO mice. Serum **a** vitamin C and **b** FGF21 levels were determined after overnight fasting by Naph-DiPy nitroxide and ELISA, respectively, in the acute cold (6 °C for 5 h)-exposed WT, SMP30-KO, and SMP30-KO mice provided vitamin C-supplemented drinking water (*n* = 7–8/group). **c** During acute cold exposure, the body temperature was measured using an infrared thermometer for small rodents (*n* = 7–8/group). For prolonged cold exposure (6 °C for 48 h), nonfasted SMP30-KO mice were given vitamin C (6 g/L) by oral gavage (approximately 7 mg/mouse/day). **d** Body weight reduction was calculated based on the difference in body weight before and after prolonged cold exposure (*n* = 6–8/group). **e** Vitamin C and **f** FGF21 levels in serum, **g** mRNA expression levels of *Fgf21* in the liver, and **h** protein levels of UCP1 in BAT were determined after prolonged cold exposure (*n* = 6–8/group). To test whether FGF21 treatment elevates thermogenic signaling, we measured **i** the mRNA expression levels of *Ucp1* and *Pgc1α* in primary adipocytes derived from the sWAT of WT mice with or without FGF21 (50 nM) treatment for 22 h (*n* = 8/group). The primary adipocytes were cultured in vitamin C-free media. Data are expressed as the mean ± SEM. *P < 0.05, **P < 0.01, and ***P < 0.001. One-way ANOVA followed by Bonferroni post hoc tests (**a**–**h** except for **c**), two-way ANOVA followed by Bonferroni post hoc tests (**c**), and two-tailed Student’s t-test (**i**).

Because FGF21 production is highly impaired in vitamin C-deficient SMP30-KO mice, we tested whether vitamin C supplementation recovers FGF21 levels and alters energy balance in SMP30-KO mice. Vitamin C water supplementation (2 g/L) recovered serum vitamin C and FGF21 levels (Fig. 4a, b), suggesting that serum vitamin C levels are closely associated with FGF21 in circulation. With the recovery of blood FGF21, the body weight notably decreased in SMP30-KO mice (Fig. 4c). In addition, serum levels of total cholesterol, glucose, and triglycerides improved in SMP30-KO mice treated with vitamin C-supplemented water (Fig. 4d–f). We next examined the effect of vitamin C on the cold tolerance of SMP30-KO mice subjected to acute and chronic cold exposure. Vitamin C supplementation recovered blood vitamin C in SMP30-KO mice to the level observed in WT mice during the acute cold challenge (Fig. 5a). In addition to the recovery of blood vitamin C, the reduced blood FGF21 and body temperature also recovered in SMP30-KO mice (Fig. 5b, c). To further investigate whether vitamin C supplementation reverses the impaired WAT browning, we performed a prolonged cold challenge test because the acute cold exposure was not sufficient for the WAT-to-BAT transition based on our preliminary studies. For the prolonged cold test, we administered vitamin C water to SMP30-KO mice by oral gavage (~7 mg vitamin C/mouse/day) to ensure sufficient vitamin C supply during the test. The prolonged cold challenge decreased body weight in WT mice by ~6.3%, but the body weight-lowering effect was much lower in SMP30-KO mice (Fig. 5d), further supporting the impaired thermogenic energy expenditure in SMP30-KO mice. Oral gavage of vitamin C fully recovered vitamin C and FGF21 in the blood (Fig. 5e and f). In addition, the mRNA expression levels of *Fgf21* in the liver of SMP30-KO mice recovered to the approximate levels observed in WT mice (Fig. 5g). These effects were accompanied by a body weight reduction of ~8.2% in SMP30-KO mice (Fig. 5d), indicating that vitamin C deficiency contributes to the dramatic decrease in FGF21 levels and thermogenic energy expenditure in SMP30-KO mice. To further test whether the recovery of FGF21 by vitamin C is associated with thermogenic signaling, the protein levels of UCP1 were measured in BAT. UCP1 protein levels were markedly reduced in the BAT of SMP30-KO mice after cold exposure but recovered to a level comparable to that of WT by vitamin C treatment (Fig. 5h). We also investigated whether FGF21 treatment elevates thermogenic signaling in primary cultured adipocytes derived from the sWAT of WT mice (Supplementary Fig. 2). When the primary adipocytes were cultured in vitamin C-free media and treated with FGF21, the mRNA expression levels of *Ucp1* and *Pgc1α* were upregulated by approximately 7- and 3-fold, respectively (Fig. 5i). Considering our data and other reports showing the effect of FGF21 on the induction of white-to-brown fat conversion and stimulation of energy expenditure^29,30^, vitamin C-mediated FGF21 recovery in SMP30-KO mice may be related to partial body temperature recovery.

### Vitamin C activates PPARα for FGF21 production

**Fig. 6:**
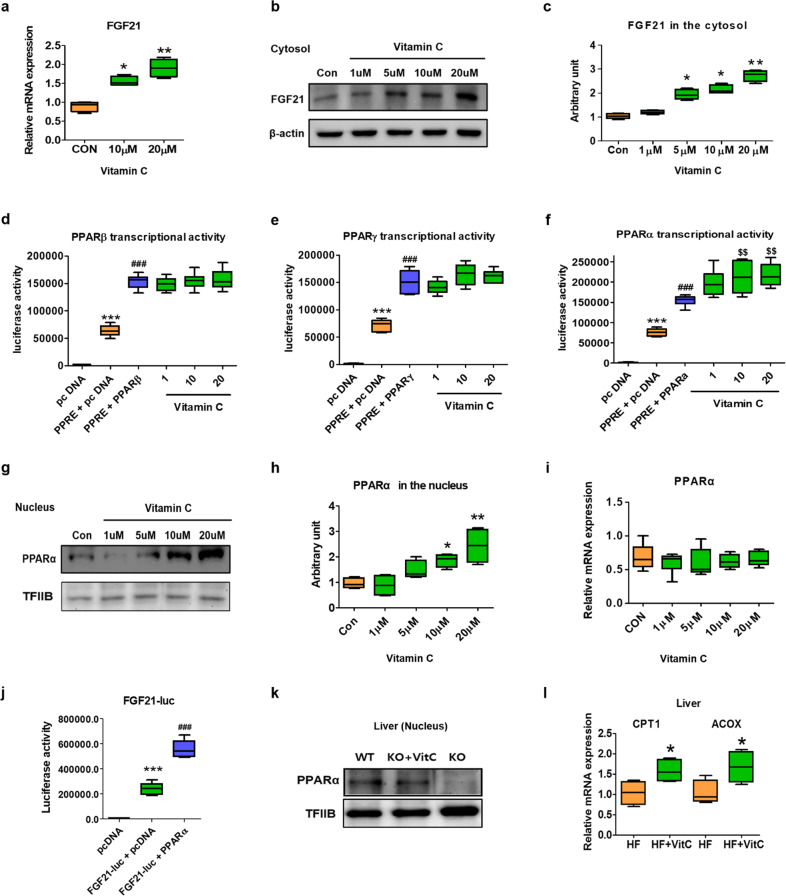
Vitamin C activates PPARα in hepatocytes. Ac2F rat hepatocytes were grown until 70–80% confluency. Twelve hours after cells were treated with vitamin C at the indicated concentration, mRNA or protein (the cytosol and nuclear fractions) was extracted. **a** mRNA and **b** protein levels of FGF21 were measured (*n* = 4/group). **c** Semiquantification of FGF21 protein levels using ImageJ. For the luciferase assay, **d**–**f** X3-PPRE-TK-LUC and PPARα, PPARβ, and PPARγ or **j** pGL3B-Fgf21-LUC expression vectors were transfected into Ac2F cells. Twenty-four hours after transfection, the cells were treated with vitamin C for 12 h. Luciferase activity was tested using the One-Glo Luciferase Assay System and a luminescence plate reader (*n* = 6/group). **g** Protein (*n* = 4/group) expression levels of PPARα in the nucleus of Ac2F cells. **h** Semiquantification of PPARα protein levels. **i** mRNA (*n* = 5–6/group) expression levels of *Pparα* in Ac2F cells. **k** The western blotting results of nuclear PPARα in the livers of WT, SMP30-KO, and SMP30-KO mice supplemented with vitamin C for 2 months. TFIIB and β-actin were used as loading controls for the nucleus and cytosol, respectively. **l** Vitamin C (2 g/L) in drinking water was supplemented to 10- to 11-week-old C57BL/6 mice with HF feeding for 3 months (*n* = 7–8/group). The mRNA levels of *Cpt1* and *Acox* were measured in the liver by qPCR. Data are expressed as the mean ± SEM. *P < 0.05 and **P < 0.01 vs. the control groups for **a**, **c**, **h**, and **l**. For **d**, **e**, **f**, and **j** (luciferase activity assays), ***P < 0.001 vs. the pcDNA group, ^###^*P* < 0.001 vs. the PPRE + pcDNA or FGF21 + pcDNA groups, and ^$$^*P* < 0.01 vs. the PPRE + PPARα group.

To determine whether vitamin C directly acts on hepatocytes for FGF21 production, we treated Ac2F hepatocytes with vitamin C and examined *Fgf21* expression. Vitamin C treatment increased *Fgf21* mRNA (Fig. 6a) and protein expression levels (Fig. 6b and c) in Ac2F hepatocytes in a concentration-dependent manner. To examine how vitamin C regulates FGF21 expression, we tested whether vitamin C controls transcription factors that have been reported to induce FGF21 transcription, including PPARα, PPARβ, and PPARγ. Luciferase assays showed that the effects of vitamin C on the transcriptional activities of PPARβ and PPARγ were not significant (Fig. 6d and e), but vitamin C elevated PPARα transcriptional activity in Ac2F hepatocytes (Fig. 6f). Consistently, western blotting showed that vitamin C increased nuclear PPARα protein levels without affecting PPARα mRNA expression levels (Fig. 6g–i), suggesting that the vitamin C-mediated increase in PPARα activity may not be dependent on the transcriptional regulation of PPARα. To investigate the PPARα agonistic effect of vitamin C in vivo, PPARα protein levels were determined in the nucleus of the liver samples obtained from WT, SMP30-KO, and SMP30-KO mice supplemented with vitamin C. Representative images showed that PPARα protein levels markedly decreased in the liver of SMP30-KO mice deficient in vitamin C, whereas vitamin C supplementation to SMP30-KO mice recovered the PPARα protein levels (Fig. 6k). In addition, vitamin C supplementation elevated the mRNA levels of *Pparα* downstream genes, including *Cpt1* and *Acox*, in the livers of WT mice (Fig. 6l). To confirm whether PPARα increases FGF21 transcription, an FGF21 luciferase assay was performed, and the data showed that PPARα transfection notably increased FGF21 transcription in Ac2F cells (Fig. 6j). These data suggest that vitamin C acts directly on hepatocytes to increase FGF21 production at least partially in a PPARα-dependent manner.

### The vitamin C-mediated increase in FGF21 may not be the sole mechanism for vitamin C-induced thermogenesis

**Fig. 7:**
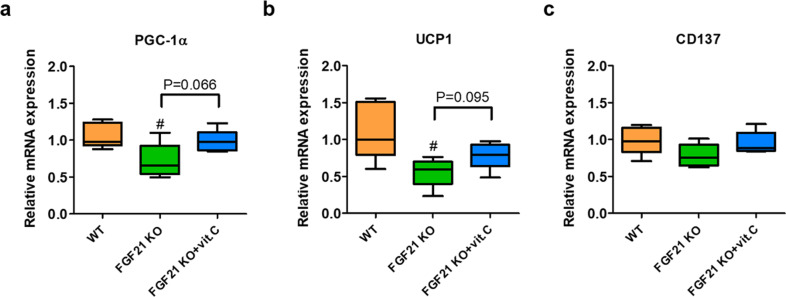
FGF21 may not be the sole mechanism underlying vitamin C-mediated thermogenesis. To test whether FGF21 is the mechanism underlying vitamin C-mediated thermogenesis, chow diet-fed male WT, FGF21-KO, and FGF21-KO mice supplemented with vitamin C (*n* = 4–5/group) were exposed to an acute cold condition (6 °C for 5 h), and the mRNA expression levels of genes related to thermogenesis were measured in the sWAT by qPCR. mRNA expression levels of **a** *Pgc1α*, **b** *Ucp1*, and **c** *Cd137*. Data are expressed as the mean ± SEM. ^#^*P* < 0.05 vs. WT mice.

To investigate whether FGF21 is the sole mechanism underlying vitamin C-mediated thermogenesis, chow diet-fed male WT, FGF21-KO, and FGF21-KO mice supplemented with vitamin C (*n* = 4–5/group) were exposed to an acute cold condition (6 °C for 5 h), and the mRNA expression levels of genes related to thermogenesis were measured by qPCR. The mRNA levels of *Pgc1α* and *Ucp1* were decreased in the sWAT of cold-exposed FGF21-KO mice, which is consistent with a previous study^29^, but no significant difference in CD137 mRNA levels was found among the groups (Fig. 7a–c). Although it was not significant, the mRNA levels of thermogenic genes, including the mRNA expression levels of *Pgc1α* and *Ucp1*, tended to be higher in the sWAT of vitamin C-supplemented FGF21-KO mice, suggesting that other mechanisms may also contribute to vitamin C-mediated thermogenesis (Fig. 7a, b).

## Discussion

**Fig. 8:**
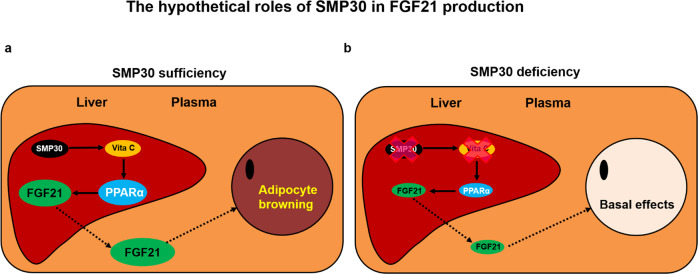
Hypothetical models describing the differences in FGF21/thermogenic signaling between normal and SMP30-deficient conditions. **a** The vitamin C-synthesizing enzyme SMP30 is highly expressed in the liver. SMP30-induced synthesis of vitamin C can activate PPARα in the livers of mice, leading to the secretion of FGF21. Then, FGF21 can go to the adipose tissue to elevate thermogenesis and regulate lipid metabolism. **b** On the other hand, SMP30 deficiency leads to impairment of the vitamin C/PPARα/FGF21 pathway in hepatocytes. Thus, the notable reduction in FGF21 in the blood contributes to the impairment in thermogenic energy expenditure in adipocytes. However, it should be noted that other mechanisms underlying vitamin C-mediated thermogenesis may also exist independent of FGF21 because vitamin C is involved in the synthesis of catecholamines, which are strong thermogenic inducers, and PPARα activated by vitamin C may also induce thermogenic signaling pathways (see the “Discussion” section for detailed information).

Despite reports that a single oral dose of vitamin C increases body temperature in humans and guinea pigs^20,21^ and that the vitamin C content increases dramatically in the BAT of rats during cold acclimation^31^, no additional studies have examined the roles of vitamin C in thermogenesis. Here, we show a previously unrecognized, yet important role of vitamin C in controlling the thermogenic activation of adipose tissues and body energy balance. As summarized in Fig. 8, the vitamin C-synthesizing enzyme SMP30 is highly expressed in the liver. SMP30-induced synthesis of vitamin C can activate PPARα in the livers of mice, leading to the secretion of FGF21. Then, FGF21 can be transported to the adipose tissue to elevate thermogenesis and regulate lipid metabolism. On the other hand, SMP30 deficiency leads to impairment of the vitamin C/PPARα/FGF21 pathway in hepatocytes. Thus, the notable reduction in FGF21 in the blood contributes to the impairment in thermogenic energy expenditure in adipocytes.

FGF21 is a well-known hormone that induces UCP1 expression and thermogenic activation of adipose tissues^29,32^. However, it is still questionable whether the FGF21-mediated elevation of thermogenic energy expenditure is the sole mechanism underlying the vitamin C-mediated beneficial effects on metabolic parameters, including the decrease in body weight, serum lipids, and fasting glucose levels, in SMP30-KO mice. FGF21 requires neither UCP1 nor beige adipocytes to induce body weight reduction and restore glucose homeostasis^33^. In addition, FGF21 deficiency has been reported to have no significant effects on energy metabolism and adipose thermogenic gene regulation during long-term cold exposure, and cold-induced browning of inguinal WAT occurs independently of FGF21^34^. In our experimental conditions, vitamin C supplementation elevated circulating FGF21 and partially recovered the impaired thermogenesis in SMP30-KO mice. In addition, FGF21 treatment in primary adipocytes cultured in vitamin C-free media significantly elevated the mRNA levels of *Ucp1*, indicating that vitamin C-mediated FGF21 elevation is a contributing factor for the partial recovery of thermogenesis in SMP30-KO mice. Nevertheless, it cannot be ruled out whether other mechanisms also contribute to the vitamin C-mediated beneficial effects. Although it was not significant, the mRNA levels of thermogenic genes tended to be higher in vitamin C-supplemented FGF21-deficient mice, suggesting that other mechanisms may also contribute to vitamin C-mediated thermogenesis. For instance, the biological functions of PPARα are broad in metabolic tissues, including the liver and adipose tissues. Our data showed that vitamin C treatment elevated PPARα transcriptional activities in hepatocytes, which possibly indicates that PPARα downstream pathways may be activated. PPARα is involved in various metabolic pathways as a sensor of nutritional status, of which it is well-known to stimulate lipid transport and *β*-oxidation of fatty acids^35^. Thus, PPARα agonists decrease lipids in blood and tissues, contributing to the improvement in metabolic syndrome^35^.

PPARα is also involved in thermogenesis. Previous studies have shown that activation of PPARα plays a critical role in the acquisition of the thermogenic capacity of BAT by inducing the expression of PGC-1α and PRDM16 in brown fat in vivo and primary brown adipocytes^36^. In addition, chronic administration of PPARα agonists in diet-induced obese mice upregulated PPARα, PGC-1α, Nrf1, TFAM, PRDM16, β3-AR, and UCP1, resulting in elevated thermogenesis accompanied by a significant body mass reduction and increased energy expenditure^37^. Although recent studies have focused on the elevation of the PPARα/FGF21 axis by vitamin C, other mechanisms, including the activation of PPARα, may likely contribute to the improved metabolic phenotypes mediated through vitamin C supplementation.

It is also possible that vitamin C may regulate thermogenic functions of adipose tissues regardless of FGF21 due to the diverse biological effects of vitamin C on oxidative stress, the synthesis of catecholamines, inflammation, embryonic stem cell differentiation, etc.^38,39^. Studies have shown that vitamin C is involved in the synthesis of catecholamines, which are neurotransmitters that are known to be major mediators of BAT thermogenic activation^40,41^. Vitamin C acts as a cofactor to optimize the activity of dopamine β-hydroxylase, the enzyme responsible for converting dopamine to norepinephrine and enhances norepinephrine biosynthesis by increasing the expression of tyrosine hydroxylase, a rate-limiting enzyme of catecholamine synthesis. Increased biosynthesis of norepinephrine activates β3-AR in adipose tissue to stimulate the kinase cascade and CREB, thereby regulating systemic thermogenesis in part through the expression of thermogenic genes, including UCP1^42^.

Therefore, follow-up studies will be necessary to reveal further mechanisms underlying the vitamin C-mediated increase in thermogenic signaling, although the current studies focused on the elevation of the PPARα/FGF21 axis by vitamin C.

Owing to deficient endogenous vitamin C production, humans cannot use vitamin C freely in environments that require thermogenesis. In addition, oral intake of vitamin C may not be enough to maintain proper vitamin C concentrations in the blood to elevate thermogenesis due to its instability and excretion through urine^43^. In this regard, SMP30-mediated endogenous vitamin C production is highly beneficial for most animals to sustain thermogenic functions in environments that require thermogenesis. In conclusion, our findings not only reveal unidentified roles for vitamin C in that it is necessary for the thermogenic activation of adipose tissues and can thereby prevent diet-induced obesity but also suggest a limitation of humans in maintaining thermogenesis, compared to animals that can synthesize vitamin C endogenously. Additional studies are necessary to examine whether defective endogenous vitamin C synthesis can explain the reduced thermogenic capacity and obesity epidemic in humans.

## Supplementary information

Supplementary figures
