# Supplementary material for: SMP30-mediated synthesis of vitamin C activates the liver PPARα/FGF21 axis to regulate thermogenesis in mice
Source: Exp Mol Med. 2022 Nov 25;54(11):2036–46. doi: 10.1038/s12276-022-00888-9 (PMC9723126; doi:10.1038/s12276-022-00888-9)
Supplement: Supplementary file 1 — Supplementary figures [file 12276_2022_888_MOESM1_ESM.pdf]

SUPPLEMENTARY INFORMATION

Supplementary Fig. 1-2

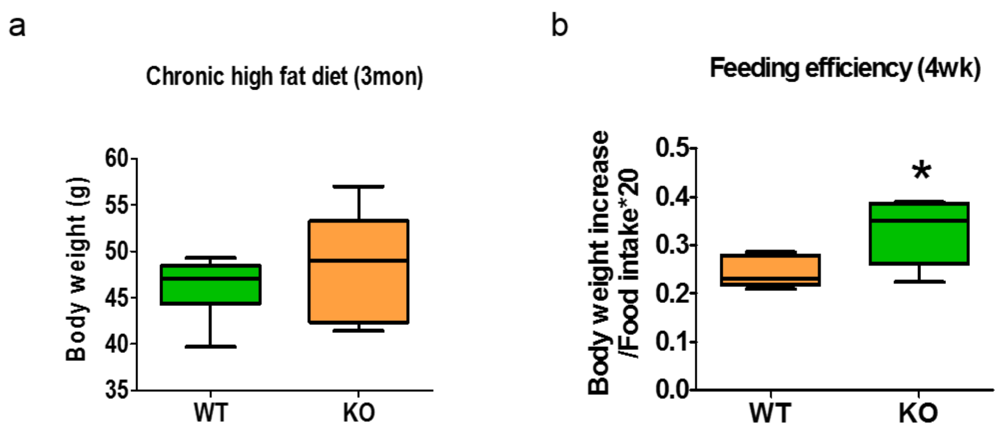

Supplementary Fig. 1. Chronic HF feeding increases feeding efficiency in SMP30 KO mice. Mice were fed an HF diet for 3 mos (n=9/group). (a) Body weight was measured after 3 mos of HF feeding and (b) feeding efficiency was calculated based on food intake and body weight gain during 4 wks of HF feeding (n=5/group). Data are represented as mean ± SEM. *P<0.05 WT vs. KO mice. Two-tailed Student's t-test (a-b)

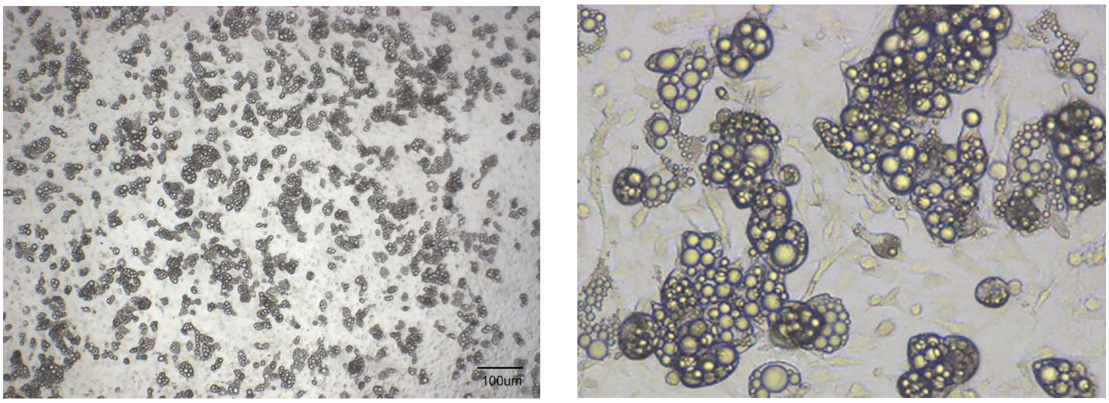

Supplementary Fig. 2. Primary cultured adipocytes from sWAT of WT mice.

6-7 wk-old male mice were used to isolate preadipocyte fraction. The cells were differentiated into adipocytes for 7 days. See methods for the detailed information. Microscopic images of differentiated adipocytes were shown.
